# Anatomical variation of the trajectory of the brachiocephalic artery encountered during parathyroid adenoma excision. A rare case report and a surgical challenge

**DOI:** 10.1016/j.ijscr.2019.04.007

**Published:** 2019-04-06

**Authors:** Maria Zarokosta, Theodoros Piperos, Dimosthenis Chrysikos, Antonios Patrinos, Dimosthenis Kakaviatos, Vassileios Kalles, Ioannis Papapanagiotou, John Tsiaoussis, Panagiotis Theodoropoulos, Theodoros Mariolis-Sapsakos

**Affiliations:** a Anatomy and Histology Laboratory, School of Nursing, University of Athens, Greece; b University Department of Surgery, General and Oncologic Hospital of Kifissia “Agii Anargiri’’, Athens, Greece; c University Department of Anatomy, Faculty of Medicine, University of Crete, Greece

**Keywords:** Brachiocephalic trunk, Anonymous artery, Anatomical variation, Aortic arch, Case report

## Abstract

**Introduction:**

The brachiocephalic trunk (BCT), also known as the “anonymous artery” constitutes the first branch of the aortic arch that bifurcates at the level of the right sternoclavicular joint into the RCCA and the RSA. Anatomical variations of the origin and the trajectory of BCT are of vital clinical significance since they constitute major risk-factors of hemorrhage when performing tracheotomy, surgeries at the anatomic area of the neck as in the presented case.

**Presentation of case:**

A 64-year-old Caucasian female proceeded to our institution with signs and symptoms of PHPT. Imaging studies performed identified a large mass localized posterior to the right thyroid lobe. During the operation, surgeons incidentally detected anterior to the trachea aberrant trajectory of the BCT. The operation was uneventful. A meticulous review of the literature was conducted as well.

**Discussion:**

Anatomical anomalies of the origin and the trajectory of BCT are vaguely described in the literature. However, these anatomic variations constitute major risk-factors of accidental bleeding and subsequent complications when performing surgeries of the thyroid and parathyroid glands, tracheotomy and invasive radiological interventions.

**Conclusion:**

Deep knowledge of such variations of the trajectory of the BCT in addition to detailed exposure of the operative field constitute the cornerstone in order surgeons to perform a safe intervention.

## Highlights

• BCT may present anatomical variations concerning its origin and its trajectory.
• Preoperative observation of these anatomical variations has vital clinical and surgical importance, since they constitute risk-factors of severe bleeding.
• Fundamentals to avoid iatrogenic injury are: (1) exposure of the trajectory and the origin of BCT, since it is quite evident that probable novel anatomic variations could be unexpectedly detected during the operation (2) good haemostasis and (3) preoperative utilization of diagnostic ultrasound.

## 1 Introduction

The brachiocephalic trunk (BCT), also known as the “anonymous artery” constitutes the first branch that emerges from the aortic arch [1,2]. The BCT at the level of the right sternoclavicular joint, typically divides into the right subclavian artery (RSA) and the right common carotid artery (RCCA) [3,4]. However, there are several documented anatomical variations of the origin and the trajectory of the BCT and it is probable that novel variations may be detected since the existing literature is quite restricted [1,5,6]. Such anatomical variations are of paramount clinical significance since they constitute major risk-factors of accidental hemorrhage when performing tracheotomy, surgeries of the thyroid and parathyroid glands, in the tumor excision of the neck and invasive radiology as well [1,3,[6], [7], [8]]. In the presented case a rare anatomical aberration of the trajectory of the BCT was incidentally detected when performing parathyroid adenoma excision in a 64-year-old female patient. The present manuscript that aims to highlight a scarce anatomical variation and its severe clinical implications has been reported in line with the SCARE criteria [9].

## 2 Case report

A 64-year-old Caucasian female with a 5-year history of recurrent episodes of nephrolithiasis that required lithotripsy proceeded to our institution with a complaint of epigastric pain and nausea the last 2 weeks in addition to fatigue and joint pain. The clinical examination was unremarkable. Subsequent laboratory investigations detected hypercalcemia (2.8 mmol/L), hypophosphatemia (0.69 mmol/L) and parathyroid hormone (PTH) (15 pmol/L).

The patient had no family history of thyroid or parathyroid disorders or history of previous radiation. No previous surgical history or commorbidities existed, apart from mild hypertension. All these findings in conjunction with the patient’s clinical presentation lead to the assumption that the patient had primary hyperparathyroidism (PHPT). Then, an ultrasound and additional Tc-99m-MIBI scintigraphy where performed, and they detected a parathyroid adenoma located posteriorly the right lobe of thyroid gland. Following these, a parathyroid adenoma surgical excision was finally scheduled.

A standard thyroid collar incision of approximately 6 cm was performed and was extended over the sternocleidomastoid muscles, approximately 2 cm above the sternal notch. While the surgeons attempted to detect and retract both the carotid artery and the jugular vein, they incidentally detected that the RCCA emerged higher from the level of the right sternoclavicular joint due to an anatomical aberration of the trajectory of the BCT, from which RCCA arises.

**Fig. 1:**
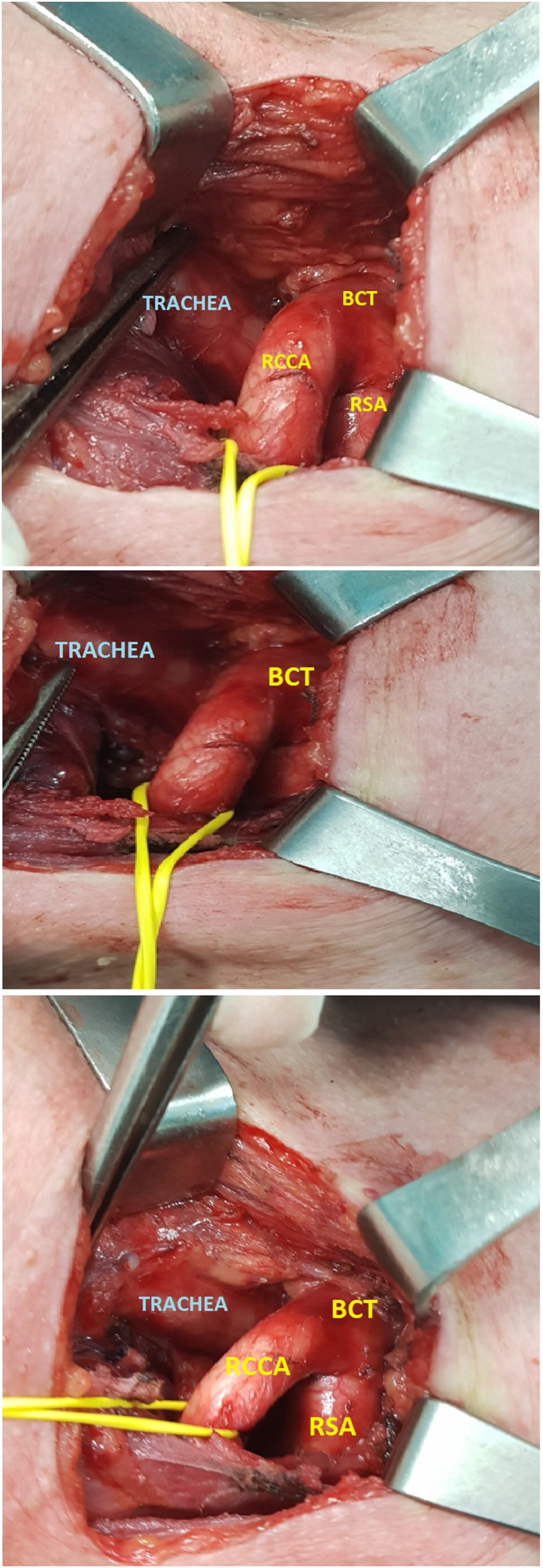
{[(Figs. 1–3)}] Thyroid collar incision. Meticulous exposure of the BCT anterior to the 3d-4th tracheal ring. The bifurcation of BCT into RCCA and RSA is detected at the level of the 4th tracheal ring.

More specifically, surgeons performed meticulous descending exposure of the RCCA and finally detected the bifurcation point of the BCT at the level of the third tracheal ring anterior to the trachea (Figs. 1–3 ). In particular, the BCT emerged as in usual fashion from the aortic arch and then ascended aberrantly to the left side of the trachea and finally divided into the RSA and the RCCA at the cervical level of the trachea. After the detailful exposure of the operative field and the gentle retraction of all the large vessels, surgeons continued as in usual fashion and the operation was uneventful, although such an anatomic variation may augment the potentiality of accidental injury and severe hemorrhage when it is not discovered preoperatively.

**Fig. 4:**
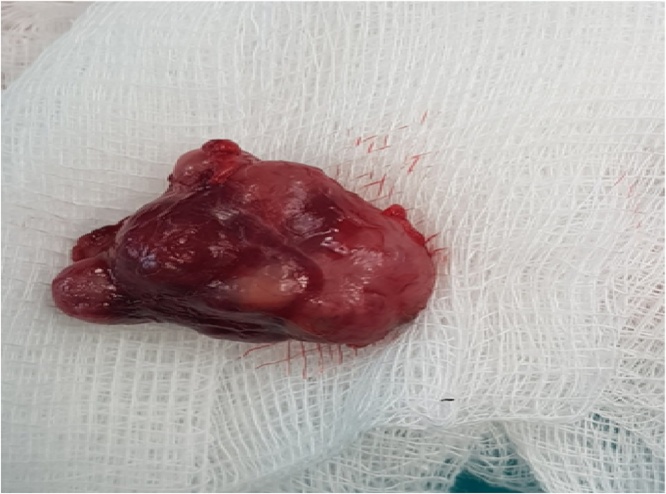
The large parathyroid adenoma removed. Despite the suspicion of malignancy, histology certified the absence of mitotic and other malignant features.

The patient was discharged with instructions the 2nd postoperative day, when the drainage placed was finally remo (Fig. 4). At the follow-up, the 7th postoperative day the patient had none complication. The histology of the mass confirmed the diagnosis of parathyroid adenoma that was composed predominantly of oxyphil cells without any malignant components. Serum calcium level was 2.69 mmol/L and iPTH 17.8 pg/mL 12 h after the operation. At the 6-month follow-up the patient had no complications.

## 3 Discussion

The brachiocephalic trunk (BCT), also known as the “anonymous artery” constitutes the first branch that emerges from the aortic arch, with an average length of 4–5 cm in adults [1,2]. Close to its origin, the anonymous artery rises to the right of the trachea, and from this point, it ascends from the posterior part of the inferior portion of the manubrium of sternum, till the level of the right sternoclavicular joint, dividing into the right subclavian artery (RSA) and the right common carotid artery (RCCA) [3,4].

The present branching type was described in 1928 by the anatomist Buntaro Adachi, as the normal pattern of bifurcation (type A) [10]. Although the current literature is quite restricted, it is documented that the BCT may present a plethora of anatomical variations concerning its origin and its trajectory, which might lead to severe complications when performing interventional procedures, surgery and angiography as well [1].

Indeed, anatomical anomalies of BCT are of paramount clinical significance during surgeries of the throat and even more important in percutaneous dilatational tracheostomy, which is an often-performed invasive technique due to its speed, simplicity and efficacy [7]. Herein, thorough knowledge of anatomical variations of great vessels of the neck is of vital significance for the surgeons since a minor accidental injury of these vessels causes a sudden massive hemorrhage [8].

Referring to these anomalies, Iterezote at al. documented a case of a human cadaver whose BCT originated from the aortic arch in front of the trachea and bifurcated into RSA and RCCA anterior to the same [6]. Comert et al. discovered the BCT originating from the aortic arch as well, but on the left side of the trachea and bifurcating on the right side of the same [3]. The most potential cause of such anatomic aberrations in the trajectory of the large blood vessels emerging from the aortic arch may be a disproportional elongation and increase of the vessel’s diameter during the embryonic life [11].

Racid et al. detected the BCT positioned in an aberrantly high level over the 2nd tracheal ring and Comert et al. over the 4th to 5th tracheal ring. It is evident that the high localization of the BCT across the trachea constitutes a major risk-factor of accidental injury in percutaneous procedures [3,8,12].

Panagouli et al. in a male human cadaver, observed the BCT giving also rise to the Left CCA from its initial trunk [1]. Finally, the point of origin of BCT typically lies to the right of midvertebral line but there are few documented cases in which the origin site was located to the left of midvertebral line and then BCT crossed upward anterior to the trachea to reach from left to right side, as in the presented case [13]. The shifting of the BCT from right to left at origin may be explained as the cranial end of aortic sac drawn out into the right and the left limb as the neck gets lengthened. In particular, the right limb forms the BCT, and the left limb becomes the part of the definitive aortic arch, located between the BCT and Left CCA [13,14].

Both surgical and invasive radiological maneuvers request an excellent knowledge of the BCT topographical anatomy and its variations. Ignorance of such anatomical aberrations may lead to serious surgical complications during procedures performed at the base of the neck, while it may also cause brain damage and even fatal hemorrhagic incidents [1].

Therefore, the preoperative observation of these anatomical variations of the trajectory of great vessels of the cervical region of the neck, including the BCT, has vital clinical and surgical importance. At present, the utilization of diagnostic ultrasound prior to percutaneous tracheotomy is a safe technique that allows the detection of the presence of anatomic variations in the trajectory of the vessels in the neck area and the subsequent avoidance of accidental injuries [15].

Finally, the fundamentals to avoid iatrogenic injury are: [1] exposure of the trajectory and the origin of BCT, since it is quite evident that probable novel anatomic variations could be unexpectedly detected during the operation [2] good haemostasis and [3] preoperative utilization of diagnostic ultrasound.

## 4 Conclusion

The presence of BCT anatomical variations, as in the presented case constitute major risk-factors when performing operation in the anatomic area of the neck, tracheostomy and interventional radiologic procedures. Indeed, serious surgical complications may be provoked including the probability of brain damage and even fatal hemorrhage. Since even an anatomic variation of minor degree may affect the outcome of such procedures, it may be concluded that surgeons’ deep knowledge in addition to meticulous operative technique are the cornerstone for safe therapeutic outcomes.

## Please state any conflicts of interest

All authors declare that there are not any competing interests.

## Please state any sources of funding for your research

There is no source of funding.

## Ethical approval

This is a Case Report for which the patient provided written informed consent. Ethical approval has also been provided by the ethical committee of the General & Oncologic Hospital of Kifissia “Agii Anarguri”.

## Consent

Written consent for the publication of this case report and accompanying images was obtained from the patient. The consent can be provided to the Editor if he asks so. The written approval of the Ethical Committee of our Institution may be provided to the Editor as well.

## Author contribution

Mariolis-Sapsakos and Zarokosta conceived of the study. Tsiaoussis was senior consultant at this case report and participated in its coordination. Kakaviatos, Kalles and Theodoropoulos contributed to the acquisition of clinical data, its analysis and interpretation and to the preparation of images. Patrinos, Zarokosta and Chrysikos contributed to literature review. Papapanagiotou, Zarokosta and Piperos contributed to the preparation of the manuscript. Mariolis-Sapsakos and Tsiaoussis contributed to the refinement of the case report. All authors have approved the final article.

## Registration of research studies

This is a Case Report and according to the Research Registry, its registration is not essential.

## Guarantor

The Guarantor who is responsible for the present case report is Theodoros Mariolis-Sapsakos. He coordinated the preparation of the case report and revised it critically for important intellectual content.

## Provenance and peer review

Not commissioned, externally peer-reviewed.
